# Minimally invasive management of biliary complications after right-lobe living donor liver transplantation: comparative analysis of two observation periods

**DOI:** 10.1097/MS9.0000000000005741

**Published:** 2026-10-23

**Authors:** Ayana Mussina, Maksat Doskhanov, Bolatbek Baimakhanov, Shokan Kaniyev

**Affiliations:** a Department of Hepatobiliary Surgery and Liver Transplantation, National Scientific Center of Surgery Named after A.N. Syzganov, Almaty, Kazakhstan; b Department of Surgery, Asfendiyarov Kazakh National Medical University, Almaty, Kazakhstan

**Keywords:** biliary complications, biliary stricture, ERCP, living donor liver transplantation, magnetic compression anastomosis, outcomes, risk factors

## Abstract

**Background:**

Biliary complications (BCs) remain the most frequent adverse events following living donor liver transplantation (LDLT). This study evaluated the incidence, patterns of severity, and outcomes of a structured, minimally invasive management strategy across two institutional periods.

**Methods:**

A retrospective analysis of 228 adult recipients of right-lobe LDLT performed between 2011 and 2025 was conducted. Patients were stratified into Period I (2011–2017) and Period II (2018–2025). The primary endpoint was successful minimally invasive management. Multivariate logistic regression analysis was performed to identify independent risk factors associated with the development of BCs.

**Results:**

Overall, 57 patients (25.0%) developed BCs. The incidence did not differ significantly between Period I and Period II (23.5% vs 25.9%; *P* = 0.69). The mean time to onset was significantly longer in Period II (6.8 ± 4.5 vs 4.2 ± 3.1 months; *P* = 0.03). Biliary strictures were the predominant complication, with complete strictures being more frequent in Period I (45% vs 11%), whereas partial strictures predominated in Period II. The success rate of minimally invasive management improved from 50.0% to 97.3% (*P* < 0.001). The rate of open surgical revision decreased from 4.7% to 0.7%, and BC-related mortality declined from 4.7% to 0%. Kaplan–Meier analysis demonstrated improved overall patient survival in Period II compared with Period I (log-rank *P* = 0.03), with five-year survival increasing from 95.3% to 100%.

**Conclusion:**

Implementation of a structured, algorithm-based, minimally invasive strategy significantly improved biliary outcomes, reduced complication severity, and eliminated BC-related mortality after right-lobe LDLT.

**Limitations:**

The retrospective single-center design and lack of long-term external validation limit generalizability. Prospective multicenter studies are required to confirm predictive factors and further refine treatment algorithms.

## Introduction

Biliary complications (BCs) remain among the most frequent and clinically significant adverse events following living donor liver transplantation (LDLT), with a reported incidence ranging from 10% to 30%, depending on graft characteristics, surgical expertise, and postoperative management strategies^[^1–6^]^. Despite substantial advances in donor selection, microsurgical reconstruction, and perioperative care, BCs continue to negatively affect graft survival and overall patient outcomes and remain a major source of long-term morbidity.

HIGHLIGHTS

- Algorithm-based minimally invasive management improved biliary outcomes after LDLT.
- Biliary stricture incidence decreased markedly between institutional periods.
- ERCP-based proactive management achieved a 97.3% success rate and zero mortality.
- Reduced cold ischemia time and prophylactic stenting enhanced graft safety.
- Institutional experience and multidisciplinary coordination drove long-term success.

The higher incidence of BCs after LDLT compared with deceased donor liver transplantation (DDLT) is primarily attributable to anatomical and technical factors. Complex biliary anatomy, smaller duct diameter, and the frequent presence of multiple bile ducts increase the complexity of biliary reconstruction^[^7–9^]^. Furthermore, limited peribiliary vascular supply and shorter duct length predispose living donor grafts to ischemic injury^[^10^]^. Biliary strictures account for approximately 70–80% of BCs and represent the predominant complication after LDLT^[^11,12^]^.

Strictures may occur early as a consequence of technical issues or ischemic injury, or later due to progressive fibrotic remodeling and inflammatory processes, often following bile leakage^[^13^]^. Clinically, they manifest as cholestasis, jaundice, or recurrent cholangitis and may ultimately result in graft dysfunction if not treated promptly^[^14,15^]^. Therefore, early detection and timely intervention are essential to prevent irreversible biliary damage.

Minimally invasive approaches constitute the current standard of care. Endoscopic retrograde cholangiopancreatography (ERCP) is considered first-line therapy, with reported success rates exceeding 80% in contemporary series^[^16–18^]^. When endoscopic access is not feasible, percutaneous transhepatic biliary drainage (PTBD) and combined rendezvous techniques provide effective alternatives^[^19^]^. For complete or refractory strictures, magnetic compression anastomosis (MCA) has emerged as an innovative minimally invasive technique that enables neo-anastomosis formation without the need for surgical revision, and has demonstrated promising long-term outcomes^[^20–25^]^.

Although improvements in outcomes over time are frequently attributed to the surgical learning curve, there is limited evidence regarding the independent contribution of structured, protocol-driven multidisciplinary management strategies to the prevention and treatment of BCs.

LDLT has been performed at our institution since 2011, accompanied by the progressive implementation of advanced endoscopic techniques, the integration of MCA into clinical practice, and the development of structured perioperative protocols. The present study evaluates the clinical impact of a standardized, algorithm-based, minimally invasive management strategy incorporating proactive biochemical surveillance, early MRCP-based detection, protocolized ERCP timing, predefined escalation criteria, and systematic integration of MCA.

The primary objective was to assess the effectiveness of this structured management algorithm in the treatment of BCs after right-lobe LDLT across two institutional periods. The secondary objective was to identify independent risk factors and to evaluate their association with long-term graft and patient outcomes. We hypothesized that implementation of a protocol-driven, multidisciplinary strategy would improve minimally invasive treatment success and reduce BC-related mortality.

## Materials and methods

### Study design

This retrospective cohort study was conducted at a single tertiary transplant center. All consecutive adult patients who underwent right-lobe LDLT between January 2011 and October 2025 were identified from the institutional database and analyzed for post-transplant BCs. The study was reported in accordance with the STROCSS 2025 criteria^[^26^]^.

### Study population

A total of 228 adult recipients of right-lobe LDLT were included. Donor and recipient demographic, perioperative, and follow-up data were retrieved from electronic medical records and operative logs.

Detailed inclusion and exclusion criteria are presented in Figure 1.

**Figure 1.**
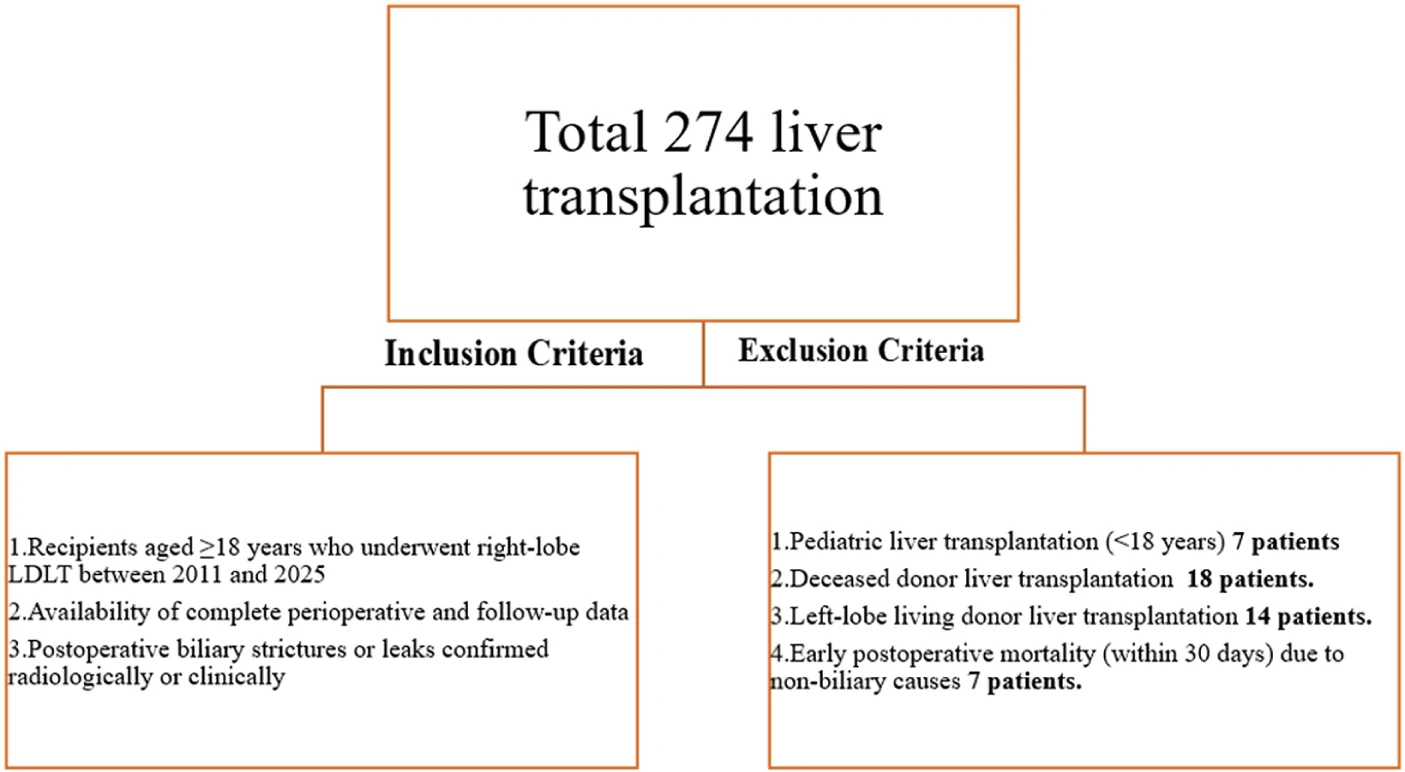
Study flowchart and patient selection process.

### Definitions

Biliary complications (BCs) were defined as postoperative biliary narrowing or leakage resulting in clinical, biochemical, or radiological evidence of obstruction or bile leak.

A biliary stricture was defined as focal anastomotic or intrahepatic duct narrowing associated with proximal duct dilatation and/or biochemical evidence of cholestasis. Radiological criteria included luminal narrowing with upstream biliary dilatation measuring ≥3 mm on MRCP or contrast-enhanced CT. Endoscopic confirmation during ERCP required a visible anastomotic narrowing necessitating balloon dilatation and/or stent placement to restore bile flow.

Minimally invasive management included:

1. ERCP with balloon dilation and/or stent placement
2. Rendezvous technique (combined percutaneous and endoscopic access)
3. Magnetic compression anastomosis (MCA) for complete or refractory strictures
4. Percutaneous transhepatic biliary drainage (PTBD)

Open surgical revision and retransplantation were considered rescue procedures following failure of minimally invasive therapy.

### Diagnostic workup and management algorithm

Postoperative surveillance included routine clinical assessment and serial laboratory testing (ALT, AST, ALP, GGT, bilirubin, coagulation profile, and inflammatory markers).

Magnetic resonance cholangiopancreatography (MRCP) was used as the primary non-invasive imaging modality for the evaluation of suspected BCs.

The structured diagnostic and therapeutic pathway is illustrated in Figure 2. The algorithm follows a stepwise escalation strategy: if a given intervention is ineffective, management proceeds to the next therapeutic level. First-line evaluation consists of MRCP followed by ERCP with balloon dilatation ± stent placement.

**Figure 2.**
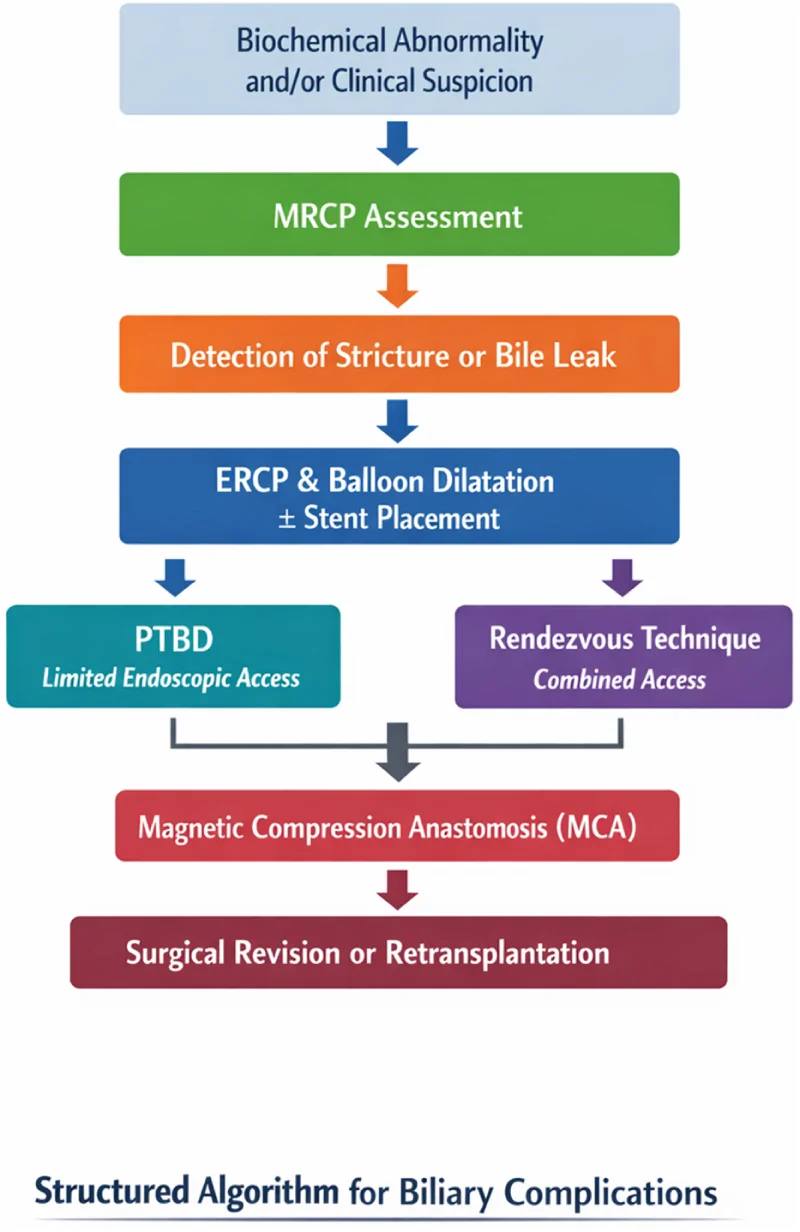
Stepwise management algorithm for biliary complications after right-lobe LDLT.

If endoscopic therapy is unsuccessful or technically infeasible, escalation to PTBD or a rendezvous technique is undertaken. Magnetic compression anastomosis (MCA) is reserved for complete or refractory strictures following the failure of standard endoscopic or percutaneous approaches. Surgical revision or retransplantation represents the final rescue option and is considered only after the exhaustion of all minimally invasive strategies.

The algorithm for the minimally invasive management of BCs is presented in Figure 2.

### Grouping by observation period

To assess temporal trends and institutional evolution, patients were stratified into two observation periods:

- **Period I (2011–2017**): Early Institutional Experience
- **Period II (2018–2025**): Multidisciplinary management with expanded minimally invasive strategies

### Variables and outcomes

The following variables were analyzed:

1. Demographic and perioperative characteristics
2. The presence of multiple bile ducts.
3. Type of biliary reconstruction
4. Timing of BCS onset
5. Type and Outcome of Intervention
6. BC-related mortality

The primary endpoint was successful minimally invasive treatment, defined as restoration of bile flow without the need for surgical revision or retransplantation.

Secondary endpoints included BC incidence, identification of independent risk factors, and BC-related mortality.

### Follow-up

Follow-up duration was calculated from the date of transplantation to the last clinical contact or death.

The median follow-up for the entire cohort was 62 months (range, 6–168). Median follow-up was 96 months (range, 48–168) in Period I and 42 months (range, 6–96) in Period II.

### Statistical analysis

Statistical analyses were performed using SPSS version 26.0 (IBM Corp., Armonk, NY, USA).

Continuous variables were expressed as mean ± standard deviation or median (interquartile range, IQR), as appropriate, and compared using Student’s t-test or the Mann–Whitney U test. Categorical variables were analyzed using the χ^2^ test or Fisher’s exact test.

Univariate logistic regression analysis was performed to identify potential risk factors for BC development. Variables with *P* < 0.10 were entered into a multivariate logistic regression model.

Candidate variables included the following.

1. Recipient's age
2. Sex
3. Presence of multiple bile ducts.
4. Cold ischemia time
5. Operative duration
6. Intraoperative blood loss
7. Type of biliary reconstruction
8. Observation period

Variable selection was based on univariate screening and clinical relevance supported by previously published risk factors. Adjusted odds ratios (ORs) with 95% confidence intervals (CIs) were calculated. A two-sided *P*-value < 0.05 was considered statistically significant.

### Ethical considerations

The study was conducted in accordance with the Declaration of Helsinki (2013 revision). Ethical approval was obtained from the Institutional Ethics Committee of the A.N. Syzganov National Scientific Center of Surgery (Protocol No. 4, dated 10 November 2023). Written informed consent was obtained from all living donors and patients before surgery and data collection.

## Results

From January 2011 through October 2025, 228 adult right-lobe living-donor liver transplants (LDLTs) were performed. Patients were categorized into Period I (2011–2017; *n* = 85) and Period II (2018–2025; *n* = 143).

### Baseline characteristics

Baseline recipient and donor characteristics were comparable between periods (Table 1).

**Table 1:** Baseline demographic, donor, and intraoperative characteristics of adult right-lobe LDLT recipients (2011–2017 vs 2018–2025) (data are presented as mean ± SD or *n* (%).

Variable	2011–2017 (*n* = 85)	2018–2025 (*n* = 143)	*P*-value	OR (95 % CI)
Recipient characteristics				
Age, years (mean ± SD)	43.2 ± 11.8	44.7 ± 10.9	0.34	—
Male, *n* (%)	45 (52.9)	62 (43.4)	0.17	1.47 (0.84–2.58)
MELD score (mean ± SD)	16.8 ± 4.1	17.6 ± 3.9	0.28	—
Donor characteristics				
Age, years (mean ± SD)	31.0 ± 7.2	28.0 ± 6.8	0.06	—
GRWR (mean ± SD)	1.05 ± 0.15	1.09 ± 0.14	0.09	—
Biliary anatomy				
Type A (single duct)	69 (81.2)	103 (72.0)	0.03	0.56 (0.33–0.96)
Type B (double ducts)	6 (7.1)	7 (4.9)	0.52	—
Type C (complex)	10 (11.8)	33 (23.1)	0.04	2.24 (1.02–4.89)
Intraoperative parameters				
Cold ischemia time, min (mean ± SD)	102.2 ± 31.5	44.0 ± 17.2	< 0.001	—
Warm ischemia time, min (mean ± SD)	31.0 ± 6.8	37.1 ± 8.2	0.02	—
Operative duration, min (mean ± SD)	823.4 ± 105.6	687.8 ± 93.2	< 0.001	—
Blood loss, mL (mean ± SD)	4387.5 ± 1216	2156.3 ± 843	< 0.001	—
Prophylactic stenting, *n* (%)	28 (32.9)	99 (69.2)	< 0.001	4.49 (2.49–8.13)

Continuous data = mean ± SD; categorical data = *n* (%). *P* < 0.05 = statistically significant. OR, odds ratio; CI, confidence interval; GRWR, graft-to-recipient weight ratio; MELD, model for end-stage liver disease.

Significant intraoperative improvements were observed in Period II.

1. Shorter cold ischemia time (102.2 ± 31.5 → 44.0 ± 17.2 min; *P* < 0.001).
2. Reduced operative duration (823.4 ± 105.6 → 687.8 ± 93.2 min; *P* < 0.001).
3. Lower blood loss (4387.5 ± 1216 to 2156.3 ± 843 mL; *P* < 0.001).

The rate of prophylactic biliary stenting increased from 32.9% to 69.2% (*P* < 0.001).

Biliary anatomy was classified according to the Huang classification system for right-lobe living donors. Type C anatomy was defined as the presence of multiple bile ducts that require separate or complex reconstruction.

Complex biliary anatomy (Type C) was more frequent in Period II (11.8% vs. 23.1%; *P* = 0.04).

The baseline demographic and clinical characteristics of the patients are presented in Table 1.

### Incidence and timing of biliary complications

Overall, 57 patients (25.0%) developed BCs.

The incidence was 23.5% (20/85) in Period I and 25.9% (37/143) in Period II (*P* = 0.69).

Biliary strictures were the predominant type, occurring in 23.5% and 6.3% of patients in Periods I and II, respectively.

The mean time to the onset of BCs was significantly longer in Period II (6.8 ± 4.5 months vs 4.2 ± 3.1 months; *P* = 0.03), indicating fewer early ischemic or technical failures.

The incidence and timing of BCs are summarized in Table 2.

**Table 2:** Incidence and timing of biliary complications after right-lobe LDLT.

Variable	2011–2017 (*n* = 85)	2018–2025 (*n* = 143)	*P*-value
Overall BCs, *n* (%)	20 (23.5)	37 (25.9)	0.69
Bile leak, *n* (%)	7 (8.2)	6 (4.2)	0.26
Biliary stricture, *n* (%)	20 (23.5)	9 (6.3)	0.0005
Leak + biloma, *n* (%)	4 (4.7)	4 (2.8)	0.48
Time to onset, months (mean ± SD)	4.2 ± 3.1	6.8 ± 4.5	0.03

BC, biliary complication; ERCP, endoscopic retrograde cholangiopancreatography; PTBD, percutaneous transhepatic biliary drainage; MCA, magnetic compression anastomosis; *P* < 0.05 = statistically significant.

### Management and therapeutic outcomes

In Period I, endoscopic management was reactive and performed only after cholestasis or cholangitis developed.

Endoscopic success was achieved in 10 of 20 patients (50%), whereas 4 (4.7%) required surgical revision, 3 (3.5%) underwent retransplantation, and 4 (4.7%) died of BC-related causes.

In Period II, a proactive multidisciplinary approach was implemented, with ERCP initiated at the first biochemical or imaging evidence of obstruction.

This strategy resulted in a 97.3% success rate (36/37; *P* < 0.001), with only one open revision (0.7%) and no retransplantations or BC-related deaths.

Minimally invasive success improved from 50.0% (95% CI, 28.4–71.6) in Period I to 97.3% (95% CI, 85.8–99.9) in Period II (*P* < 0.001).

he outcomes of minimally invasive and surgical management are presented in Table 3.

**Table 3:** Outcomes of minimally invasive and surgical management of biliary complications after right-lobe LDLT [data are presented as mean ± SD or n (%)].

Variable	2011–2017 (*n* = 85)	2018–2025 (*n* = 143)	*P*-value
Patients with BCs treated minimally invasively, *n* (%)	20 (23.5)	37 (25.9)	0.69
Type of primary intervention			
Endoscopic (ERCP ± stent), *n* (%)	6 (30.0)	18 (48.6)	0.01
Percutaneous (PTBD), *n* (%)	4 (20.0)	3 (8.1)	0.04
Rendezvous/MCA combined, *n* (%)	3 (15.0)	14 (37.8)	0.04
Surgical interventions			
Open surgical revision, *n* (%)	4 (4.7)	1 (0.7)	0.07
Liver retransplantation, *n* (%)	3 (3.5)	0 (0)	0.06
Therapeutic outcomes			
Effectiveness of minimally invasive therapy, *n* (%)	10 (50.0)	36 (97.3)	< 0.001
Time from diagnosis to intervention, days (mean ± SD)	7.3 ± 4.1	3.6 ± 2.2	0.002
Mean ERCP sessions (mean ± SD)	2.3 ± 1.1	1.6 ± 0.8	0.01
Hospital stay, days (mean ± SD)	24.6 ± 8.3	17.9 ± 6.1	0.001
BCs-related mortality, *n* (%)	4 (4.7)	0 (0)	0.03

BC, biliary complication; ERCP, endoscopic retrograde cholangiopancreatography; PTBD, percutaneous transhepatic biliary drainage; MCA, magnetic compression anastomosis; *P* < 0.05 = statistically significant.

### Severity of biliary complications

To better characterize the clinical impact beyond incidence, patterns of severity were analyzed.

The severity pattern of biliary strictures is presented in Table 4.

**Table 4:** Severity pattern of biliary strictures.

Variable	Period I (*n* = 20 strictures)	Period II (*n* = 9 strictures)
Anastomotic	17 (85%)	7 (78%)
Non-anastomotic	3 (15%)	2 (22%)
Complete obstruction	9 (45%)	1 (11%)
Partial narrowing	11 (55%)	8 (89%)

Data are presented as *n* (%). Percentages are calculated within each period (Period I: *n* = 20 strictures; Period II: *n* = 9 strictures). Anastomotic and non-anastomotic strictures refer to the anatomical location of narrowing. Severity was classified as complete obstruction or partial narrowing based on cholangiographic findings.

Complete strictures were substantially more frequent in Period I.

Early technical leaks predominated in Period I.

Complete obstruction occurred in 45% (95% CI, 25.8–65.8) of strictures in Period I compared with 11% (95% CI, 2.0–43.5) in Period II.

The severity pattern of bile leaks is presented in Table 5.

**Table 5:** Severity Pattern of Bile Leaks.

Variable	Period I (*n* = 7 leaks)	Period II (*n* = 6 leaks)
Early (<3 months)	5 (71%)	2 (33%)
Late (>3 months)	2 (29%)	4 (67%)
Leak with biloma	4 (57%)	4 (67%)

Data are presented as *n* (%). Percentages are calculated within each period (Period I: *n* = 7 leaks; Period II: *n* = 6 leaks). Early leaks were defined as occurring within 3 months after transplantation, and late leaks as occurring beyond 3 months. Biloma was diagnosed radiologically and/or intraoperatively.

Although complex graft anatomy increased in Period II, severe postoperative obstruction decreased.

The anatomical distribution of BCs is shown in Table 6.

**Table 6:** Anatomical distribution of biliary complications.

Variable	Period I (*n* = 20 BCs)	Period II (*n* = 37 BCs)
Single-duct involvement	15 (75%)	30 (81%)
Multiple-duct involvement	5 (25%)	7 (19%)

Data are presented as *n* (%). Percentages are calculated within each period (Period I: *n* = 20 BCs; Period II: *n* = 37 BCs).

Single-duct and multiple-duct involvement refer to the number of affected bile ducts identified on cholangiography or MRCP.

### Magnetic compression anastomosis (MCA)

According to Table 3, rendezvous/MCA combined procedures were performed in:

- 3 patients were in Period I.
- 14 patients in Period II

Among these are the following:

- Period I: 2 MCAs, 1 rendezvous
- Period II: 8 MCA, 6 rendezvous

Integration of MCA into the treatment algorithm in Period II contributed to a marked increase in minimally invasive success (97.3%) and the elimination of BC-related mortality.

Clinical success was 50% (95% CI, 9.5–90.5) in Period I and 100% (95% CI, 67.6–100) in Period II, as detailed in Table 7.

**Table 7:** Outcomes of magnetic compression anastomosis.

MCA Variable	Period I (*n* = 2)	Period II (*n* = 8)
Technical success	2 (100%)	8 (100%)
Clinical success	1 (50%)	8 (100%)
Recurrence	1 (50%)	0
Procedure-related complications	0	1 (12.5%) mild cholangitis
Median follow-up (months)	52	28

Data are presented as *n* (%) or median values where appropriate (Period I: *n* = 2; Period II: *n* = 8). Technical success was defined as successful magnet approximation and recanalization of the biliary tract. Clinical success was defined as resolution of cholestasis and radiological patency without the need for additional surgical intervention. Follow-up duration is reported as median months after the procedure.

### Long-term outcomes

Following successful minimally invasive therapy, biliary patency was maintained in 90% of patients, and the recurrence rate was 14.3%. All recurrent cases were successfully managed with repeat ERCP.

Kaplan–Meier survival analysis demonstrated improved overall patient survival in Period II compared with Period I (log-rank *P* = 0.03). As shown in Figure 3, five-year overall survival was **95.3% in Period I** and **100% in Period II**.

**Figure 3.**
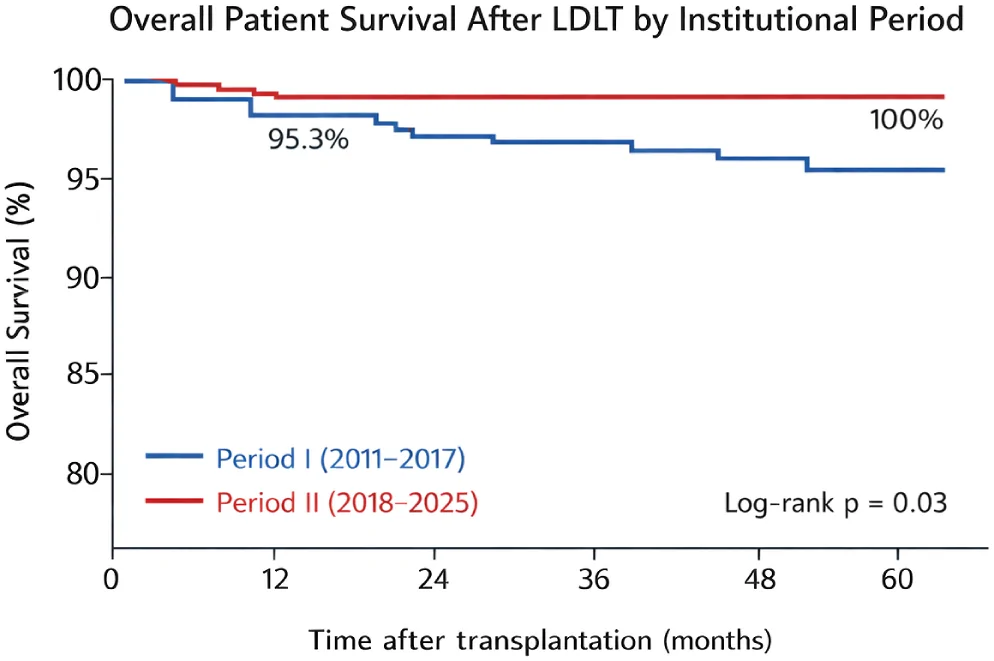
Kaplan–Meier analysis of overall patient survival after right-lobe living-donor liver transplantation (LDLT) by institutional period.

These findings confirm the durable benefit of a structured, algorithm-based, minimally invasive management strategy and reflect the cumulative institutional learning effect over time.

## Discussion

### Principal findings

BCs remain among the most frequent and clinically significant adverse events following liver transplantation, particularly in LDLT, where multiple bile duct anastomoses and limited peribiliary microcirculation increase susceptibility to ischemic injury^[^1–4^]^. Despite continuous technical refinement, the reported rates of BCs remain between 10% and 30% worldwide^[^2,6,16,22^]^.

In this 14-year comparative analysis, the implementation of a structured, algorithm-based, minimally invasive management strategy was associated with improvements in clinical severity and outcomes rather than in overall incidence. Although the overall incidence of BCs did not change significantly (23.5% vs 25.9%; *P* = 0.69), the clinical impact was substantial: minimally invasive success increased from 50% to 97.3%, the need for surgical revision was nearly eliminated, and BC-related mortality was reduced to zero.

### Evolution of severity and timing

Beyond incidence alone, a notable shift in complication severity was observed. Period I was characterized by a higher proportion of complete anastomotic strictures and early bile leaks, whereas Period II was dominated by partial strictures identified at earlier stages of development.

The significantly later onset of BCs in Period II carries important clinical implications. Early strictures and bile leaks are typically associated with technical factors or acute ischemic injury and tend to follow a more aggressive course. In contrast, later-onset strictures are often progressive and are detected during routine biochemical or imaging surveillance, allowing intervention before complete obstruction develops.

Importantly, the delayed presentation observed in Period II did not reflect postponed diagnosis. Rather, it was associated with a markedly lower proportion of complete strictures (45% vs. 11%), a reduced need for surgical revision, and complete elimination of BC-related mortality. This pattern suggests improved anastomotic integrity, reduced early ischemic injury, and more effective postoperative surveillance, resulting in complications that were milder and more amenable to minimally invasive therapy.

These observations are consistent with findings by Yamaguchi *et al*^[^27^]^, who highlighted the importance of preserving microvascular integrity, as well as Zhao *et al*^[^28^]^ and Goumard *et al*^[^29^]^, who demonstrated that internal stenting and intraoperative cholangiography reduce the risk of ischemic cholangiopathy and bile leakage.

### Impact of algorithm-based early intervention

A key determinant of improved outcomes was the transition from reactive to proactive management. During 2011–2017, ERCP was performed primarily after clinical deterioration. From 2018 onward, intervention was initiated in response to early biochemical abnormalities or MRCP findings, before overt clinical decline.

Wang *et al*^[^30^]^ demonstrated that imaging-based prediction can limit secondary intrahepatic involvement. Our findings support this concept: reduced latency between detection and intervention was associated with a substantial decline in complete strictures and surgical revisions.

In addition, the significant reduction in cold ischemia time (from 102 to 44 minutes, *P* < 0.001) likely contributed to improved biliary integrity, in line with Karakoyun *et al*^[^31^]^, who identified ischemia duration as an independent predictor of stricture formation.

### Role of magnetic compression anastomosis

The integration of magnetic compression anastomosis (MCA) into the treatment algorithm further expanded minimally invasive options for complete or refractory strictures. Although early experience was limited, systematic incorporation of MCA during the later period resulted in high technical and clinical success rates and eliminated the need for retransplantation in these complex cases.

The effectiveness of hybrid radiological–endoscopic collaboration, as described by Iwasawa *et al*^[^32^]^ together with structured drainage strategies reported by Kalisvaart *et al*^[^33^]^, supports the concept that multidisciplinary algorithmization enhances long-term therapeutic durability.

### Comparison with contemporary multicenter data

Outcomes observed during Period II are comparable with those reported by high-volume transplant centers. Park *et al*^[^34^]^ and Mayer *et al*^[^35^]^ reported favourable long-term outcomes of staged endoscopic and salvage approaches, while Yoon *et al*^[^22^]^ reported a BC rate of 14% with no mortality under structured endoscopic management.

Importantly, our data suggest that structured institutional protocolization – rather than surgical learning alone – was central to outcome improvement. This distinction is crucial, as many longitudinal series attribute better outcomes primarily to technical maturation over time.

### Institutional learning and system-level evolution

Over the 14-year study period, our center evolved into a fully integrated multidisciplinary program uniting transplant surgeons, hepatologists, radiologists, and endoscopists. Reduced latency between detection and intervention, clearly defined decision pathways, and systematic imaging surveillance were pivotal contributors to improved outcomes.

Figure 4 illustrates the evolution of the institutional management strategy between Period I and Period II and demonstrates in Figure 4, demonstrating the transition from a reactive management approach to a structured, proactive algorithm.

**Figure 4.**
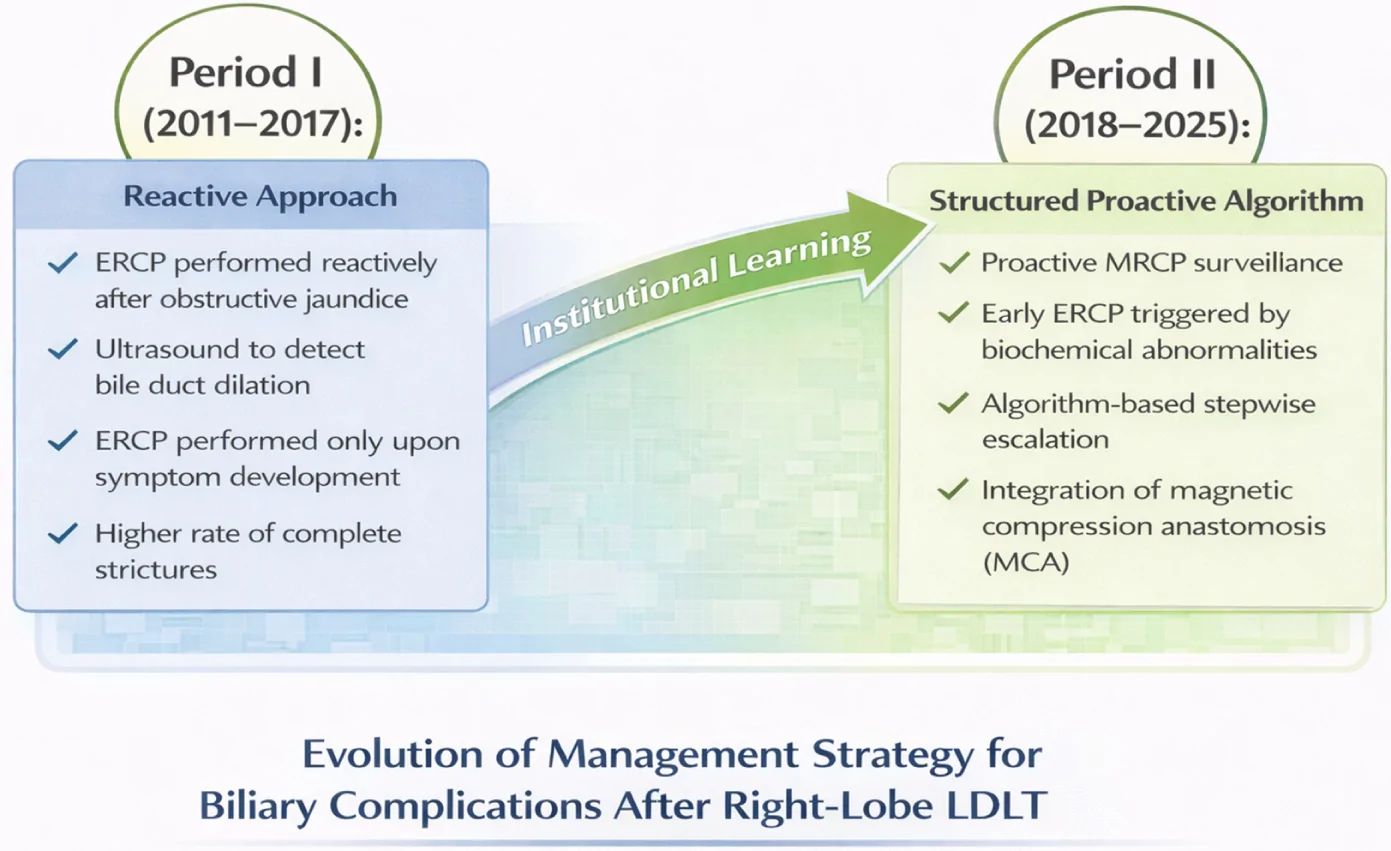
Evolution of the management strategy for biliary complications after right-lobe LDLT.

A comparison between the reactive approach used during Period I (2011–2017) and the structured, proactive algorithm implemented in Period II (2018–2025) reflects institutional learning and optimization of minimally invasive management.

### Clinical implications

The clinical relevance of our findings extends beyond a reduction in incidence. Earlier detection, a lower proportion of complete obstruction, and elimination of BC-related mortality indicate that structured surveillance combined with timely, minimally invasive intervention can modify the natural history of post-transplant biliary injury.

Such an approach may be particularly valuable for emerging transplant programs seeking to reduce the need for surgical revision and retransplantation while maintaining long-term graft function.

### Limitations

This study has several limitations:

1. **Single-center design and learning-curve bias:** The 14-year study period included institutional maturation of surgical expertise and multidisciplinary coordination, which may have influenced outcomes independently of the biliary management algorithm.
2. **Changes in donor selection criteria:** Over time, a higher proportion of complex biliary anatomy was accepted, potentially affecting complication patterns and limiting the strict comparability between periods.
3. **Evolution of perioperative and postoperative management:** Improvements in cold ischemia time, anesthesia, critical care protocols, and increased use of prophylactic biliary stenting may have contributed to differences in outcomes beyond the structured biliary strategy.
4. **Advances in imaging technology:** Greater availability and improved quality of MRCP during the later period may have enabled the earlier detection of subclinical strictures, influencing the observed shift toward less severe obstruction patterns.

Therefore, although the findings support the benefit of a structured minimally invasive strategy, external validity remains limited, and multicenter prospective validation is warranted.

## Conclusion

BCs remain a major challenge after LDLT. Improved techniques and proactive endoscopic management have significantly reduced the severity of biliary complications and the need for surgery. A structured, multidisciplinary approach has enhanced safety, long-term outcomes, and overall success in managing BCs.

All authors have read and approved the final manuscript.

## Data Availability

The datasets generated and/or analyzed during the current study are available from the corresponding author on reasonable request.
